# Novel molecular hepatocellular carcinoma subtypes and RiskScore utilizing apoptosis-related genes

**DOI:** 10.1038/s41598-024-54673-x

**Published:** 2024-02-16

**Authors:** Menggang Zhang, Shuijun Zhang, Wenzhi Guo, Yuting He

**Affiliations:** 1 Department of Hepatobiliary and Pancreatic Surgery, The First Affiliated Hospital of Zhengzhou University, Zhengzhou, 450052 China; 2 Key Laboratory of Hepatobiliary and Pancreatic Surgery and Digestive Organ Transplantation of Henan Province, The First Affiliated Hospital of Zhengzhou University, Zhengzhou, 450052 China; 3 Open and Key Laboratory of Hepatobiliary and Pancreatic Surgery and Digestive Organ Transplantation at Henan Universities, Zhengzhou, China; 4 Henan Key Laboratory of Digestive Organ Transplantation, Zhengzhou, China

**Keywords:** Cancer models, Cancer models

## Abstract

Hepatocellular carcinoma (HCC) is the third leading cause of global cancer-related deaths. Despite immunotherapy offering hope for patients with HCC, only some respond to it. However, it remains unclear how to pre-screen eligible patients. Our study aimed to address this issue. In this study, we identified 13 prognostic genes through univariate Cox regression analysis of 87 apoptosis-related genes. Subsequently, these 13 genes were analyzed using ConsensusClusterPlus, and patients were categorized into three molecular types: C1, C2, and C3. A prognostic model and RiskScore were constructed using Lasso regression analysis of 132 significant genes identified between C1 and C3. We utilized quantitative polymerase chain reaction to confirm the model’s transcript level in Huh7 and THLE2 cell lines. Both molecular subtypes and RiskScores effectively predicted patients benefiting from immunotherapy. Cox regression analysis revealed RiskScore as the most significant prognosis factor, suggesting its clinical application potential and providing a foundation for future experimental research.

## Introduction

According to the newest global cancer statistics, China leads in both new cancer case number and related deaths^1^. Liver cancer, a major contributor to cancer-related deaths, ranks sixth in incidence and third in mortality worldwide. Male liver cancer mortality rate even reaches second place globally^1^. The most common pathological subtype of liver cancer is hepatocellular carcinoma (HCC), accounting for approximately 80% of cases^2,3^. While traditional treatments have mainly focused on surgical treatment, immunotherapy has emerged as a novel viable therapeutic method. However, only partial HCC patients responded to immunotherapy, and applying immunotherapy to all patients without proper selection may lead to poor results and financial burden^4,5^. Hence, identifying the HCC subtype suitable for immunotherapy is crucial.

The immune system constantly detects and eliminates various anomalies within the human body, including mutated tumor cells, dead cells, and apoptotic cells. This process is known as immune monitoring. During tumor initiation, immune infiltration plays a key role in inhibiting and killing tumor cells^6^. Lymphocytes, primarily T lymphocytes (accounting for approximately 80% of them) are the main drivers of anti-tumor effects^7,8^. Immune infiltration is positively correlated with improved prognosis and reduced metastatic rates in HCC^8^. Moreover, Zheng et al. also reported that patients with HCC and higher lymphocyte infiltration exhibit a longer post-surgery survival^9^.

Tumor-infiltrating lymphocytes stimulate programmed cell death including apoptosis, pyroptosis, necroptosis, and ferroptosis in tumor cells^10^. Among these cell death types, apoptosis is the most well-researched. It has been found to exert tumor-suppressive or tumor-promoting effects in different cellular states^11^. While apoptosis has traditionally been considered a critical tumor suppression mechanism induced by lymphocyte stimulation and nutrient deprivation in tumor tissue, Morana et al. have revealed that apoptosis can increase genome instability and the likelihood of tumor cell occurrence^12^. Additionally, apoptosis in the tumor cell population can promote cancer and immunotherapy resistance by influencing the tumor microenvironment (TME), primarily composed of immune cells^12^. Consequently, establishing the HCC subtype based on the associated apoptosis characteristics could help in selecting patients responsive to immunotherapy.

In our study, we developed a prognostic model utilizing prognostically significant constituents within the apoptosis pathway. We further explored the functional characteristics, benefits of immunotherapy, and sensitivity to chemotherapy drugs for these different molecular subtypes. To validate the reliability of our prognostic model, we used data from the GSE78220 and GSE135222 immunotherapy datasets. Furthermore, we refined our prognostic model's ability to predict survival utilizing Cox regression analysis.

## Results

### The expression profile and SNV situation of apoptosis-related genes in HCC patients

**Figure 1:**
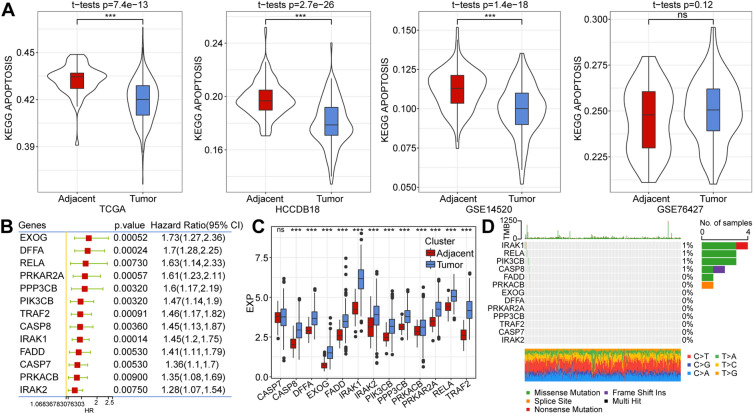
(**A**) Comparative single-sample gene set enrichment analysis scores of the Kyoto Encyclopedia of Genes and Genomes “APOPTOSIS” pathway in tumors vs. adjacent tumors in four datasets. (**B**) Single factor Cox analysis of apoptosis-related genes with prognostic significance in The Cancer Genome Atlas dataset. (**C**) Differential expression analysis of apoptosis-related genes with prognostic significance between tumors and adjacent cancers. (**D**) Mutation analysis of genes with significant prognostic significance on cell apoptosis in tumors and adjacent cancers.

We utilized ssGSEA to compute KEGG_APOPTOSIS signaling pathway scores in multiple datasets (TCGA, HCCDB18, GSE14520, and GSE76427) and compared the scores between tumor and para-tumor samples. In all datasets except GSE76427, the KEGG_APOPTOSIS signaling pathway scores were higher in para-tumor samples than in tumor samples (Fig. 1A). Subsequently, single-factor Cox analysis was carried out for the 87 apoptosis-related genes in the TCGA data. Among these, 13 genes were significantly associated with the prognosis of patients with HCC, being considered risk factors for the pathology (Fig. 1B). We examined the differential expression of these 13 genes in tumor and para-tumor tissues, observing that 12 among them exhibited expression differences and all were over-expressed in tumor tissues (Fig. 1C). Additionally, we assessed the mutation frequency of these 13 genes in HCC, finding generally low mutation frequencies, with four genes having a 1% mutation rate, while the remaining genes exhibited no mutations (Fig. 1D).

### Molecular typing of prognostic significance genes based on the APOPTOSIS pathway

**Figure 2:**
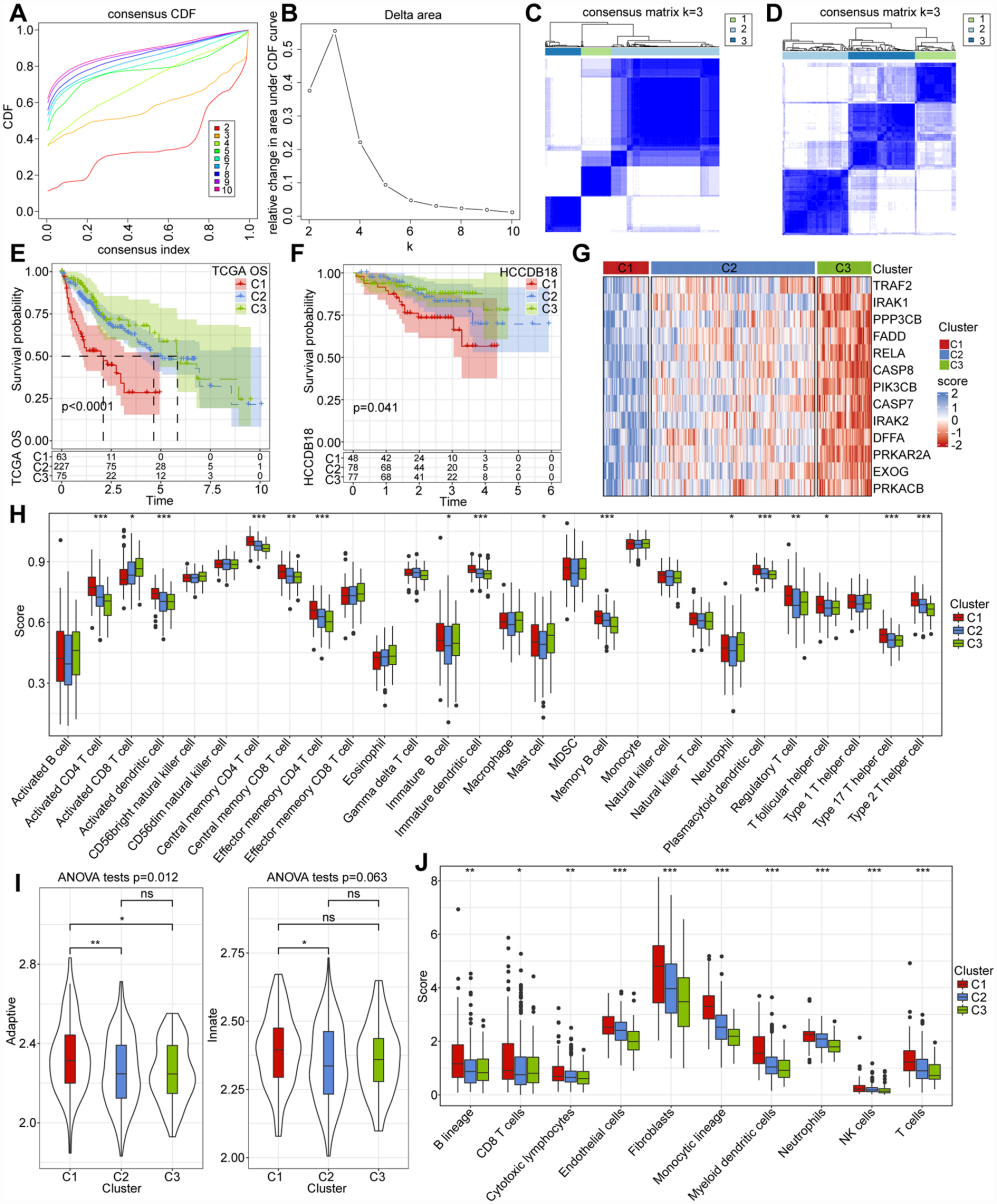
(**A**,**B**) CDF and CDF Delta area curves for The Cancer Genome Atlas (TCGA) samples. The x-axis represents category number (k), while the y-axis displays percentage change in the area under the CDF curve. (**C**) Consensus cluster heatmap for TCGA samples (k = 3). (**D**) Consensus cluster heatmap for HCCDB18 samples (k = 3). (**E**,**F**) Prognostic KM curves for TCGA and HCCDB18 datasets. (**G**) Heatmap of genes in the TCGA dataset. (**H**) Distribution of 28 immune scores in TCGA subtypes. (**I**) Distribution of innate and acquired immunity in TCGA subtypes. (**J**) Distribution of 10 immune scores in TCGA subtypes (R, version 4.3.2, https://cran.r-project.org/).

We used 13 apoptosis-related prognostic genes for consensus clustering (ConsensusClusterPlus) of TCGA samples, utilizing the cumulative distribution function (CDF) to determine the optimal number of clusters^13^. The CDF Delta area curve revealed that selecting k = 3 yielded a relatively stable clustering outcome (Fig. 2A,B). Thus, we chose k = 3 to identify three HCC molecular subtypes (Fig. 2C). The same method applied to the HCCDB18 dataset yielded similar results (Fig. 2D). Subsequent survival analysis of three subtypes revealed the C3 subtype exhibited the most favorable prognosis, while the C1 subtype had the least favorable prognosis (Fig. 2E,F). And aforementioned 13 apoptosis-related genes showed higher expression levels in the C3 subtype, compared to the C1 subtype (Fig. 2G). Furthermore, we assessed immune infiltration among the different subtypes using the ssGSEA method, analyzing immune cell gene profiles from the literature^14,15^. The results indicated that the C1 subtype with a poor prognosis in the TCGA dataset had a higher immune score, whereas the C3 subtype with a better prognosis had a lower immune score (Fig. 2H–J).

### Identification and functional analysis of differentially expressed genes between C1 and C3 subtypes

**Figure 3:**
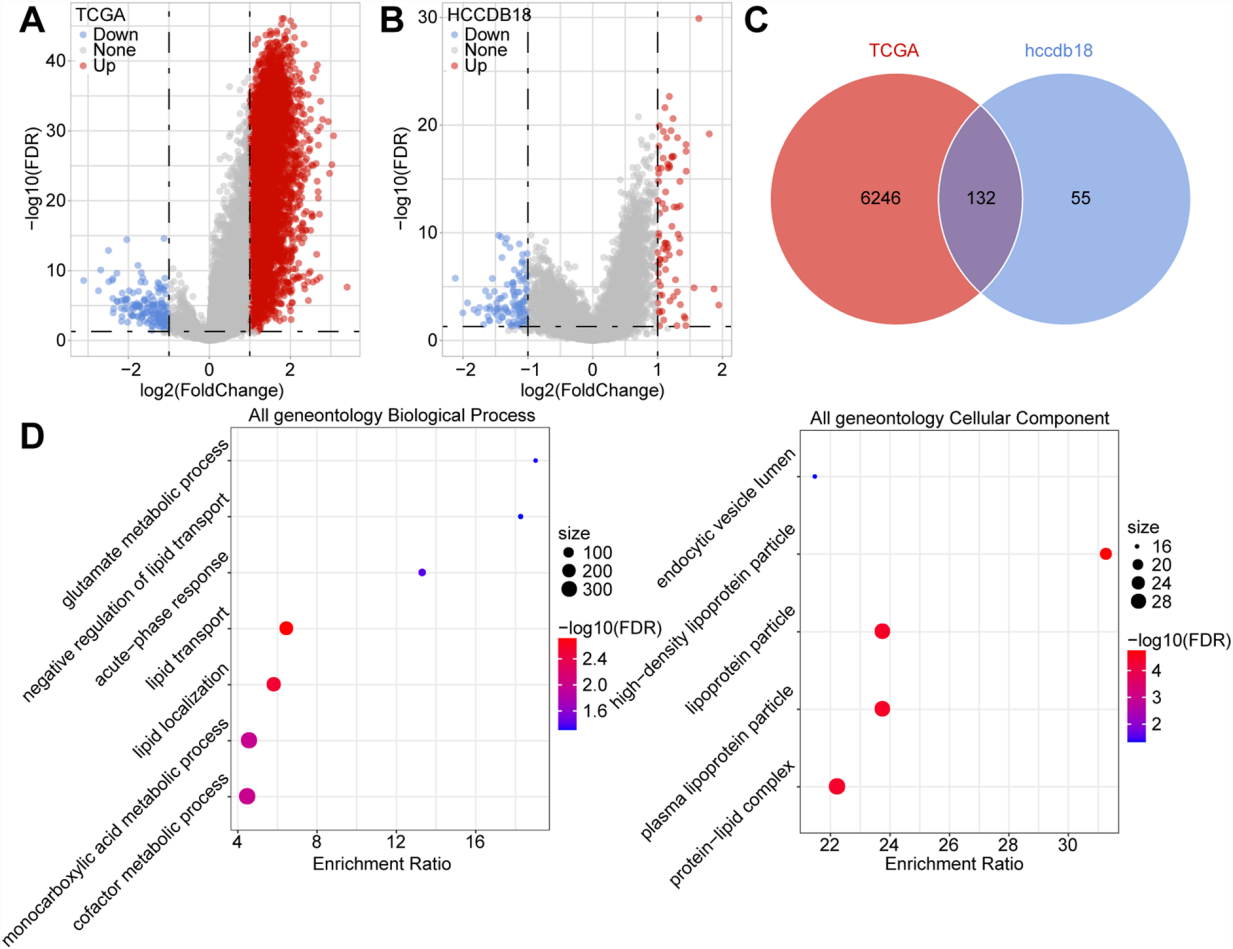
(**A**,**B**) Volcano maps showcasing the differentially expressed genes of the C1 and C3 subtypes in both The Cancer Genome Atlas (TCGA) and HCCDB18 datasets. (**C**) Intersection of differentially expressed genes between TCGA and HCCDB18 datasets. (**D**) GO functional enrichment analysis of the genes intersecting between TCGA and HCCDB18 datasets (R, version 4.3.2, https://cran.r-project.org/).

To understand the molecular basis of the immune score differences between the C1 and C3 subtypes, we employed the limma package to analyze differentially expressed genes between C1 and C3 in both TCGA and HCCDB18 datasets. In the TCGA dataset, 6378 genes displayed differential expression, with 6209 being upregulated and 169 downregulated (Fig. 3A). Additionally, in the HCCDB18 dataset, 187 genes exhibited differential expression, including 109 upregulated and 78 downregulated genes (Fig. 3B). Subsequently, we performed GO functional enrichment analysis on the 132 genes shared between the discrepant genes from TCGA and HCCDB18 utilizing the R package WebGestaltR (V0.4.4) (Fig. 3C). The results indicated a significant association between metabolism and these differentially expressed genes (Fig. 3D).

### Construction of prognostic models

**Figure 4:**
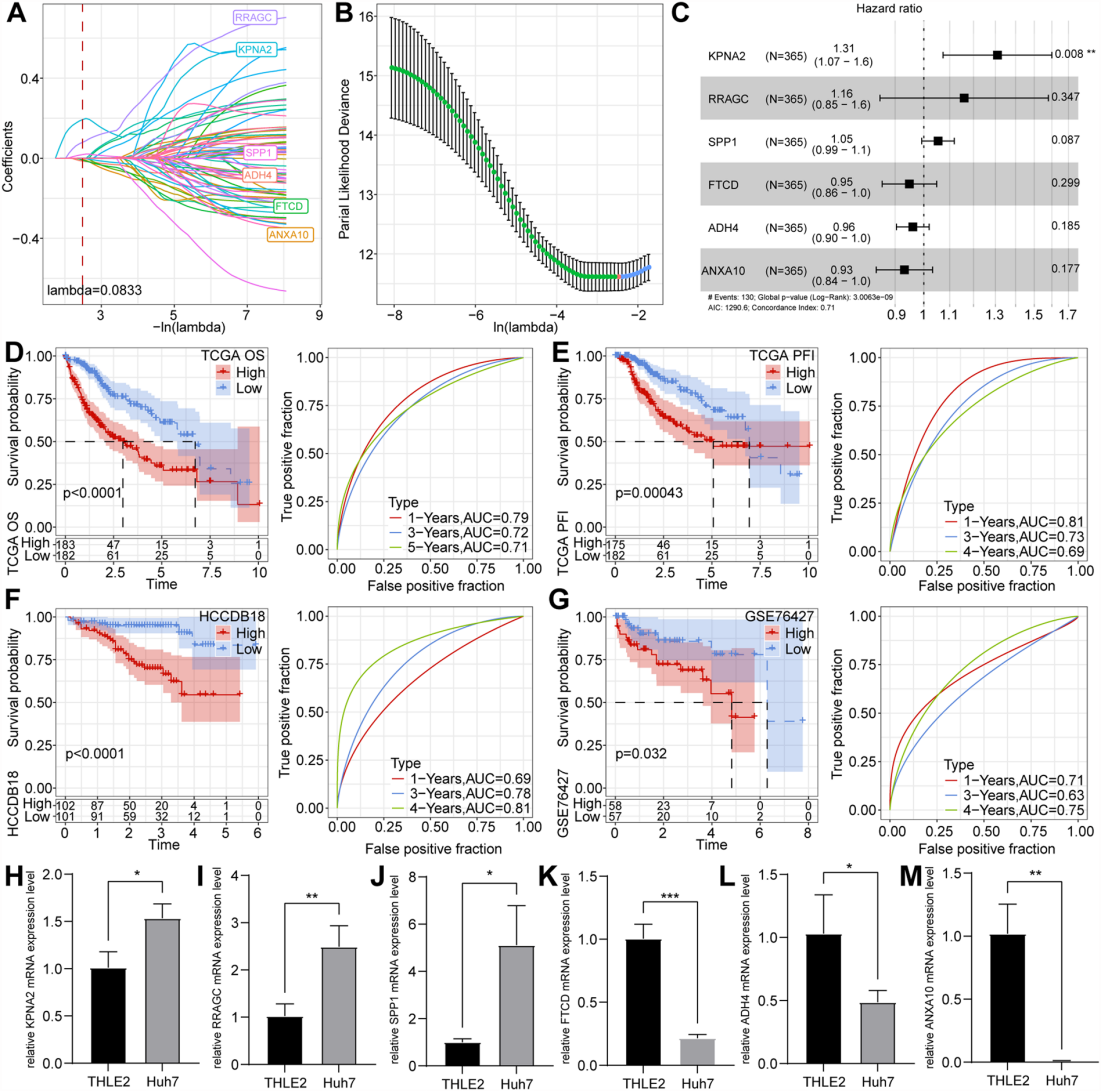
(**A**) Trajectory of independent variable changing with γ. (**B**) Confidence interval under γ. (**C**) Multi-factor analysis results for risk model genes. (**D**,**E**) Kaplan Meyer (KM) survival and receiver operating characteristic (ROC) curves for RiskScore in different time subtypes of The Cancer Genome Atlas dataset. (**F**,**G**) Verification of KM survival and ROC curves for RiskScore in the HCCDB18 and GSE76427 validation datasets. (**H**–**M**) The differential expression of six genes (KPNA2, RRAGC, SPP1, FTCD, ADH4, and ANXA10) in THLE2 and Huh7 cell lines.

We analyzed 132 differentially expressed genes using univariate Cox regression, identifying 76 significant genes (P < 0.05). We refined the model using Lasso regression through tenfold cross-validation and analyzed the confidence interval for each γ parameter, as illustrated in Fig. 4A^16^. The optimal state was found at γ = 0.0833 (Fig. 4B). Therefore, we selected 6 genes for further analysis when γ = 0.0833. A multivariate Cox regression analysis determined the risk coefficient for each of these 6 genes (Fig. 4C). The RiskScore formula was the following:

$${\text{RiskScore}} = 0.{27} \times {\text{KPNA2}} + 0.{148} \times {\text{RRAGC}} + 0.0{52} \times {\text{SPP1}} - 0.0{53} \times {\text{FTCD}} - 0.0{41} \times {\text{ADH}}$$

$${4} - 0.0{71} \times {\text{ANXA1}}0.$$

We conducted qPCR to assess the expression of the above-mentioned six genes in THLE2 and Huh7 cell lines. qPCR results closely mirrored the risk coefficient trends of the prognostic model. In Huh7 liver cancer cell, Karyopherin subunit alpha 2 (KPNA2), ras-related GTP binding C (RRAGC), and Secreted phosphoprotein 1 (SPP1) were overexpressed compared to THLE2 cell, while formimidoyltransferase cyclodeaminase (FTCD), alcohol dehydrogenase 4 (ADH4), and Annexin A10 (ANXA10) were down-regulated compared to normal liver cell (Fig. 4H–M). We also validated in 8 pairs of clinical samples and obtained results with the same trend (Supplementary Fig. 1A–F).

### Validation of clinical prognosis models

Using the surv_cutpoint function from the survminer package, we determined the optimal cutoff value for classifying patients into high-risk or low-risk groups. We plotted survival curves for prognostic analysis using the KM method and determined the significance of the differences using a log-rank test. RiskScores were calculated for each patient based on the RiskScore formula. Furthermore, we utilized the timeROC package to conduct ROC analysis for RiskScore prognosis classification.

We analyzed the training dataset to assess the efficiency of prognosis prediction for 1 year, 3 years, and 5 years and obtained high areas under the curve. By using the RiskScore as a cutoff, we divided samples into high- and low-risk groups generating a KM curve (Fig. 4D,E) that differentiated the two groups (p < 0.001). Patients with a higher RiskScore displayed lower overall survival (OS) rates or shorter progression-free intervals in the training dataset compared to patients with a lower RiskScore. To validate the reliability of clinical prognosis models based on risk-related gene signatures, we examined cases from liver cancer databases such as HCCDB18 and GSE76427. The RiskScore was computed for each patient in the validation set using the same techniques, and the results demonstrated similar outcomes as in the training set. High RiskScore was associated with a poorer prognosis, while low RiskScore indicated a more favorable prognosis (Fig. 4F,G).

### Analysis of immunotherapy TIDE in molecular subtypes and risk groups

**Figure 5:**
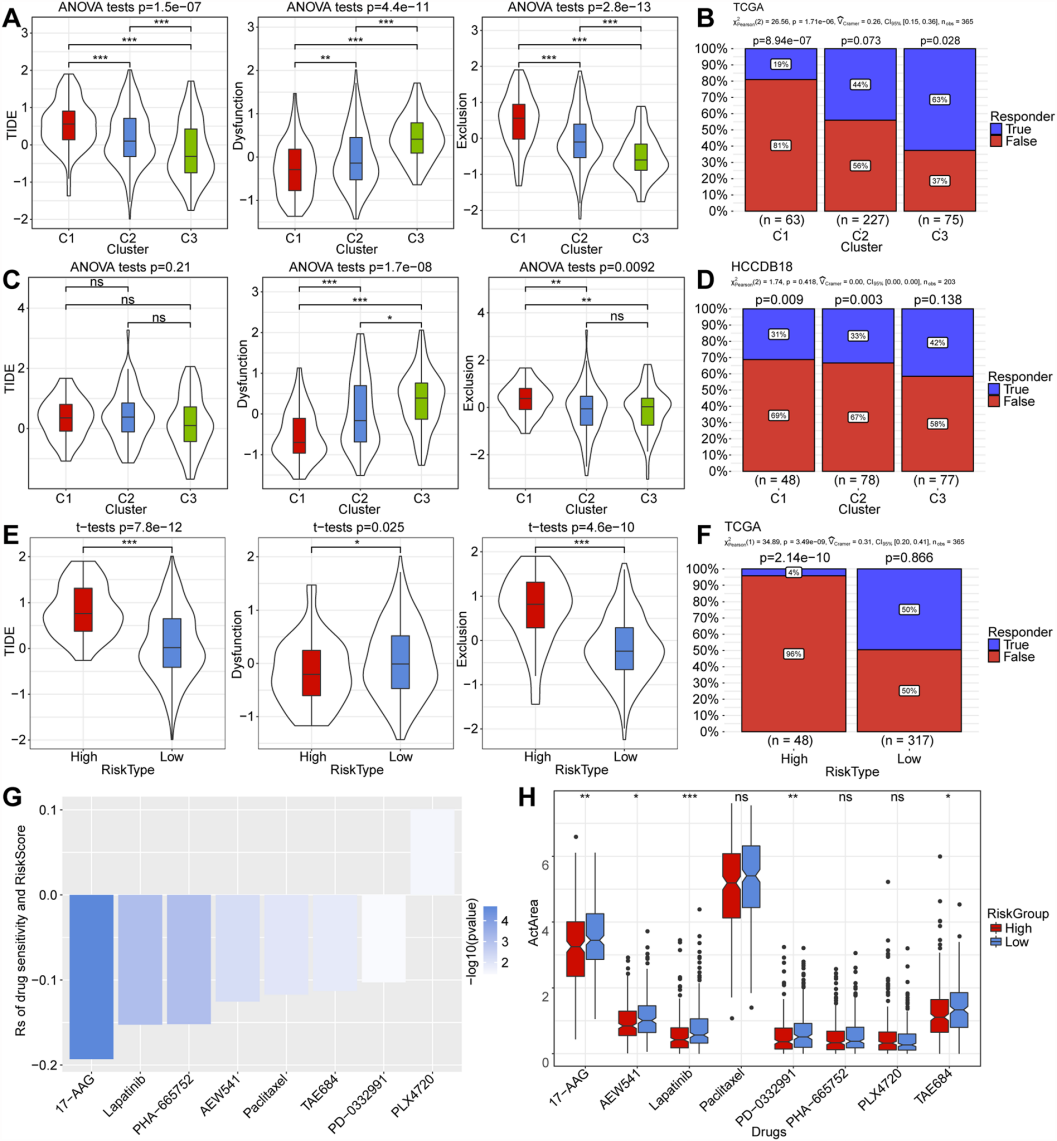
(**A**,**C**) Differences in TIDE scores were observed among various subtypes in The Cancer Genome Atlas (TCGA) and HCCDB18 datasets. (**B**,**D**) The proportion of patients benefiting from immunotherapy across different subtypes in the TCGA and HCCDB18 datasets. (**E**) Differences in TIDE scores between high and low-risk groups in the TCGA dataset. (**F**) The proportion of patients benefiting from immunotherapy in high and low-risk categories of the TCGA dataset. (**G**) The correlation between TCGA dataset RiskScore and drug sensitivity in the Encyclopedia of Cancer Cell Lines (CCLE) database. (**H**) The CCLE database utilizes the RiskScore model to determine the distribution of ActArea for each drug.

We evaluated the clinical outcomes of immunotherapy in our high-risk and low-risk groups utilizing TIDE software. A higher TIDE prediction score suggests a greater probability of immune escape, indicating reduced potential benefit from immunotherapy^16^. As illustrated in Fig. 5A, the TIDE score is lowest in the C3 group in the TCGA dataset, indicating a higher possibility of benefiting from immunotherapy. The C1 subtype has a lower Dysfunction score but a higher Exclusion score (Fig. 5A). Comparing immunotherapy benefits among subtypes, we found that subtype C3 had a higher benefit proportion (Fig. 5B). Similar results were observed in the HCCDB18 dataset (Fig. 5C,D). In the TCGA dataset, the low-risk group had a lower TIDE score, indicating a higher possibility of benefiting from immunotherapy. The high-risk group exhibited a lower Dysfunction score but a higher Exclusion score (Fig. 5E,F).

### Sensitivity analysis of chemotherapy drugs in high-risk and low-risk groups

We obtained drug sensitivity data from the Encyclopedia of Cancer Cell Lines for 1037 cancer cells (http://sites.broadinstitute.org/ccle). We used ActArea in liver cancer cell lines as an indicator of drug response and assessed the correlation between drug sensitivity and RiskScore using Spearman correlation analysis (Fig. 5G). We identified significant correlations with |Rs|> 0.1 and FDR < 0.05. Subsequently, we compared drug sensitivity differences between different risk groups, screening 8 drugs, including 17-AAG, lapatinib, PHA-665752, AEW541, paclitaxel, TAE684, PD-0332991, and PLX4720. Among them, the drugs 17-AAG, AEW541, lapatinib, PD-0332991, and TAE684 showed significant differences, with low-risk patients exhibiting greater sensitivity to these five medications (Fig. 5H).

### Prognostic model in pan-cancer

**Figure 6:**
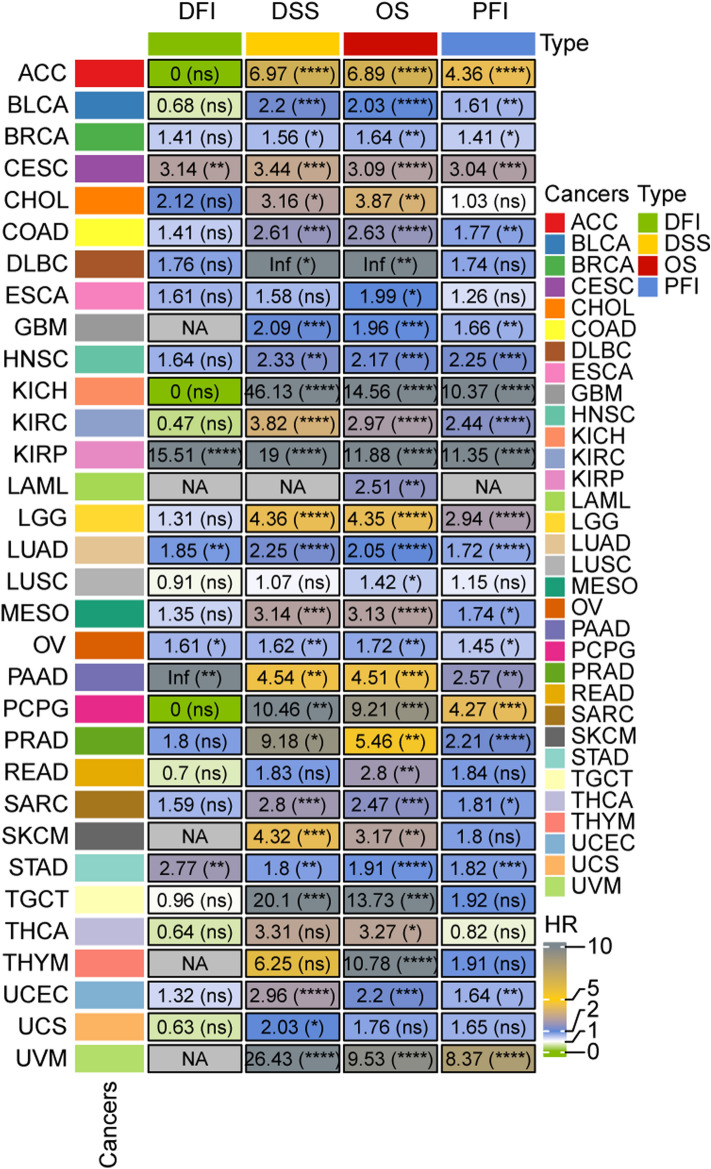
RiskScore analysis of prognosis at different time points in pan-cancer. The numbers inside represent the hazard ratio (HR) value; * in parentheses below represents the log-rank P value of HR. * represents P-values < 0.05, ** represents P-values < 0.01, *** represents P-values < 0.001, **** represents P-values < 0.001, and the gray ‘NA’ indicates that there is either no corresponding survival time and status information available for the tumor or that HR values cannot be calculated.

We acquired expression data and relevant clinical information for 32 different tumor types from the TCGA database. Utilizing our RiskScore model, we calculated RiskScores for each cancer type and established optimal cutoff values based on OS and status. Subsequently, we analyzed the survival curves for groups with high and low RiskScores, calculating hazard ratios HR and corresponding P-values across OS, progression-free interval (PFI), disease-free interval (DFI), and disease-specific survival (DSS) and status, and visualizing them. The results were as follows: (1) In terms of OS, our RiskScore revealed significant differences between high and low RiskScores in 31 cancer types, with high RiskScore indicating significantly worse prognosis than low RiskScore; (2) In terms of PFI, we observed significant differences between groups with high and low RiskScores for 21 cancer species, where high RiskScore was associated with poorer prognosis than low RiskScore; (3) DFI analysis indicated significant differences in six cancer types, with high RiskScore correlating with worse prognosis than low RiskScore; (4) In the case of DSS, our RiskScore demonstrated significant differences between high and low RiskScores in 26 cancer types, with high RiskScore indicating worse prognosis than low RiskScore (Fig. 6).

### Prognostic model comparison in immunotherapy datasets

**Figure 7:**
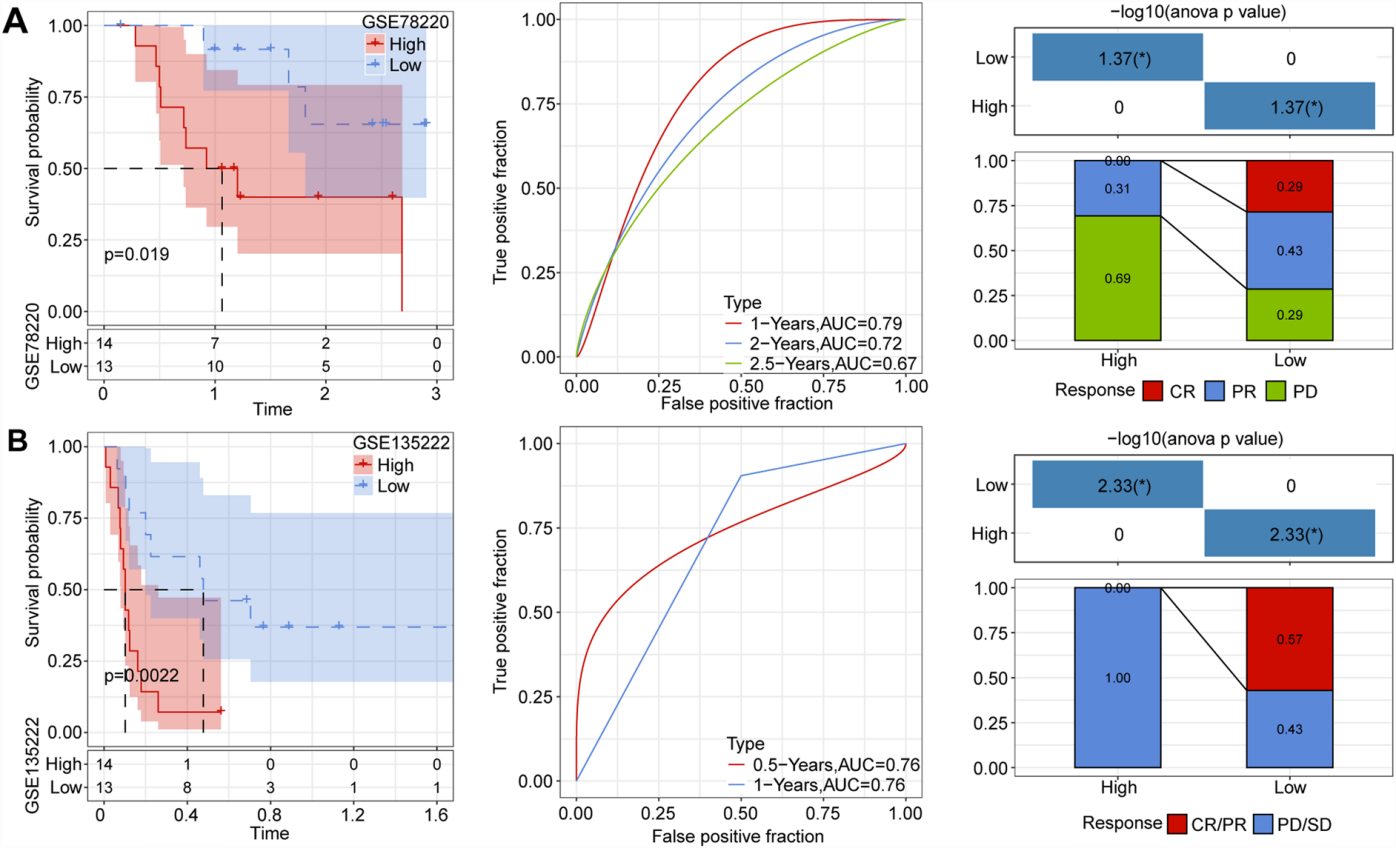
(**A**,**B**) RiskScore survival, receiver operating characteristic (ROC) curve, and immunotherapy distribution in datasets GSE78220 and GSE135222.

We analyzed the immunotherapy-treated datasets GSE78220 and GSE135222 using our approach to calculate RiskScores. Subsequently, we predicted survival curves based on Riskscore and identified the optimal cutoff point for generating KM curves. The results indicated significant differences between our Riskscore subgroups, with a higher incidence of progressive disease/stable disease observed in the high-risk group compared to the low-risk group. This aligns with the TIDE analysis outcomes confirming that high-risk grouping in the context of immunotherapy is associated with relatively lower benefits (Fig. 7A,B).

### RiskScore combines clinical and pathological characteristics to enhance prognosis models and survival prediction

**Figure 8:**
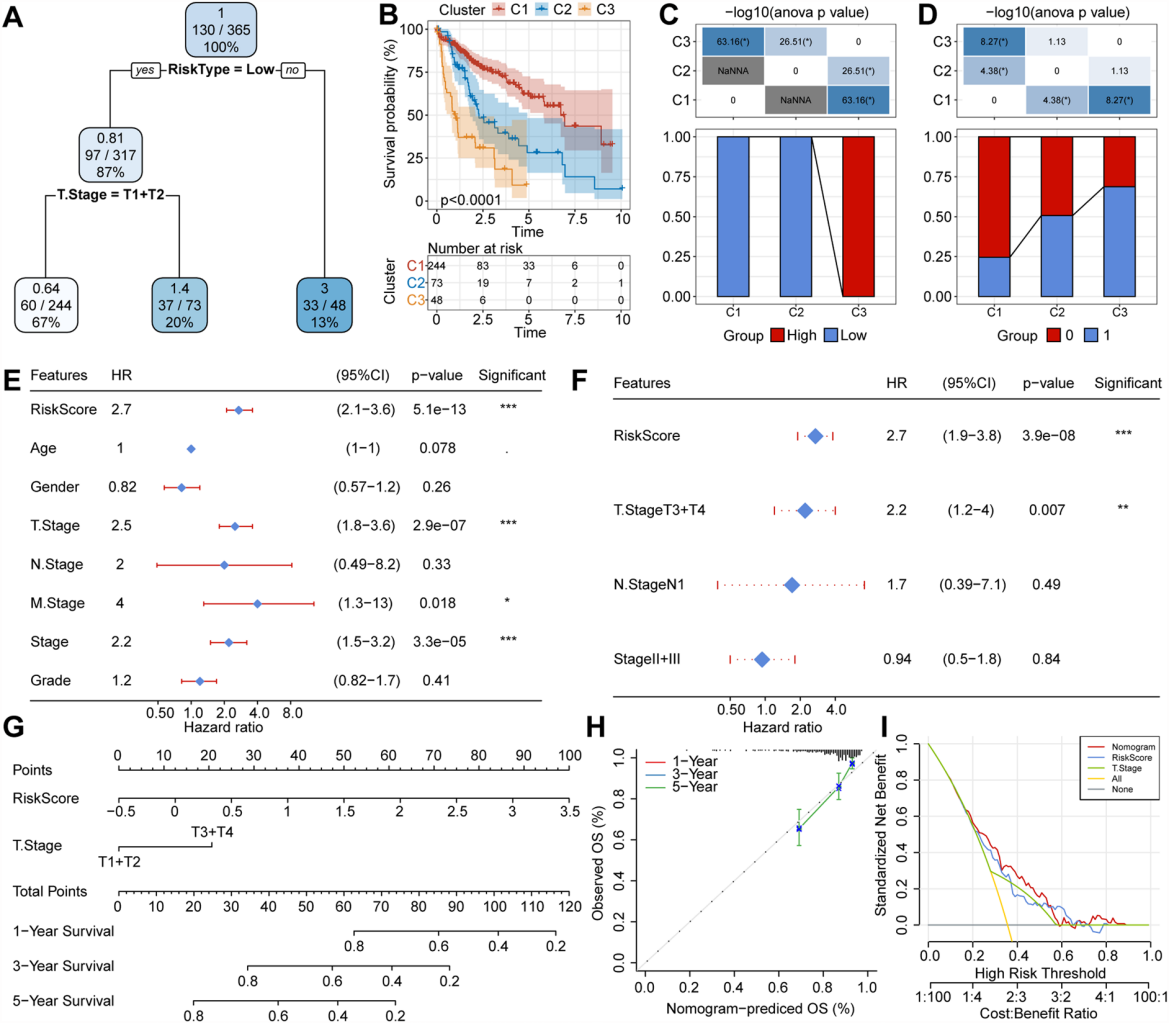
(**A**) A survival decision tree was developed using patient annotations including RiskScore, disease stage, gender, and age to optimize risk stratification. (**B**) Significant differences in overall survival for liver hepatocellular cancer were observed among the three risk subgroups. (**C**,**D**) Comparative analysis was conducted between different groups. (**E**,**F**) Single factor and multi-factor Cox analysis of RiskScore along with clinical pathological features. (**G**) Column chart model. (**H**) Calibration curves of the column chart for 1, 3, and 5 years. (**I**) The decision curve of a column chart.

We generated a decision tree using data from patients with liver cancer in the TCGA-LIHC dataset, incorporating age, gender, TNM staging, grade, and RiskScore as input variables. This analysis revealed the persistent significance of RiskScore and T Stage as key features in the final decision tree, effectively categorizing patients into three distinct risk subgroups. Furthermore, RiskScore emerged as a significant predictor of disease outcome (Fig. 8A). These risk subgroups exhibited significant variations in the OS rates (Fig. 8B), with C3 risk subgroup exclusively comprising patients with high RiskScore, while C1 and C2 subgroups exclusively included those with low RiskScore (Fig. 8C). Furthermore, differences in survival status were observed among patients in various risk subgroups (Fig. 8D). Cox regression analysis, considering RiskScore alongside clinical and pathological features, emphasized that RiskScore held the utmost significance as a prognostic factor (Fig. 8E,F). To quantitatively assess risk and survival likelihood for patients with liver cancer, we integrated RiskScore with clinical characteristics, generating a column chart (Fig. 8G). The results underscored RiskScore as the most influential factor in survival prediction.

Additionally, we assessed the predictive accuracy of the model using calibration curves. The predicted calibration curves for the three calibration points (1, 3, and 5 years) closely aligned with the standard curve, indicating excellent predictive performance (Fig. 8H). Furthermore, we assessed the model's reliability using DCA revealing that both RiskScore and the Nomogram outperformed the extreme curve. Compared to other clinical and pathological characteristics, the Nomogram and RiskScore exhibited the most potent predictive ability for survival^16^ (Fig. 8I).

## Discussion

In cancer, immunotherapy has advantages including good prognosis and minimal side effects. However, the response of patients with HCC to immunotherapy falls short of expectations, limiting its application^17,18^. Due to its unique physiological characteristics including material metabolism, detoxification, synthesis, and transformation, the liver exhibits a TME distinct from that of other organs. Thus, the liver itself, due to its own characteristics, is easily able to cope with external conditions for intervention, including immunotherapy. The immunotherapy may lose effectiveness after metabolization by the liver. Therefore, identifying HCC patients with low potential for immune escape, and high immunotherapy benefits is crucial.

After in-depth study, it is revealed that the risk factors of HCC will lead to hepatocyte death and Severe inflammatory response^19^. The cytokines produced by activated immune cells promote liver cell transformation and activate anti apoptotic pathways, leading to the growth of HCC^20^. This is also an important reason why we need to construct a new subtype of hepatocellular carcinoma from the apoptotic pathway of cells.

Moreover, Granito et al. found that regulatory T cells (Tregs) inhibited the ability of activated T cells and contributed to the progression and metastasis of HCC. They noticed that most of HCC patients have hepatitis virus infection, which are much more than autoimmune liver diseases^21^. There is already sufficient evidence to suggest that treg plays an important promoting role in the pathogenesis of HCC patients with viral hepatitis, especially CD4 + CD25 + Tregs, and immune checkpoint inhibitors (ICIs) targeting these immune cells can have therapeutic effects on HCC^21^. This is consistent with our research findings. The enrichment of Tregs cells in C1 subtype is significantly higher than that in C3 subtype, and the prognosis of C1 subtype is also poor (Fig. 2H).

The tumor immune microenvironment (TIME) is closely related to the occurrence and development of tumors, and T cells are an important component of TIME, playing an important role in acquired immunity^22^. CD8 cytotoxic T cells are the main tumor killing cells and have non-specific killing effects on most tumors^23^. This confirms our research findings (Fig. 2H). Abnormal inflammation may enhance the activation of innate immune responses, including the recruitment of neutrophils and dendritic cells. And these innate immune cells in C3 subtype are all less than those in C1 subtype. Previous studies have found that innate immune cells play a promoting role in the occurrence, postoperative recurrence, and immune therapy resistance of HCC^24–26^.

Apoptosis plays a very important role in the tumor immune process. While some research has explored immune-related HCC molecular subtypes, none has focused on constructing subtypes from apoptosis-related genes^27,28^. Due to the intricate role of apoptosis in tumor outcomes and its close correlation to immune response, creating HCC subtypes based on apoptosis-related genes can more effectively differentiate patients with HCC, identifying those benefiting the most from immunotherapy. We obtained sample data from databases such as TCGA, HCCDB18, GSEA database, and revealed through ssGSEA and Cox regression analysis that apoptosis related pathways play a very important role in HCC development. Subsequently, we identified 13 marker genes significantly associated with liver cancer prognosis. Some of these genes have been reported to be involved in the development of tumors. Shi et al. found through case–control studies on cervical cancer patients and normal controls that the Polymerphisms in Caspase-7 (CASP7) can increase the risk of cervical cancer by regulating programmed cell death^29^. Moreover, in leukemia, interleukin 1 receptor-associated kinase (IRAK1) can regulate interferon (INF) -γ signal transduction, which induces myoid derived suppressor cells, leading to immune escape in tumors^30^. Thus, it is very promising to further search for immune subtypes based on these prognostically significant genes.

Then we defined three liver cancer subtypes using consensus clustering. We found that there were significant differences in patient outcomes among the three subtypes, especially between C1 and C3 subtypes. And the expression differences of the 13 prognostic significant genes mentioned above are also more significant. Subsequently, we noticed that there were obvious differences in immune cell infiltration and immune status between the C1 and C3 subtypes. Overall, C1 has a higher immune score while C3 has a lower immune score. Moreover, there were significant differences in adaptive immunity, while there was no remarkable difference in innate immunity. It suggests that there is no significant change in innate immunity during the process of liver cancer resistance to immunotherapy, while adaptive immunity plays a crucial role in this process. And this is consistent with the previously mentioned characteristic that the liver itself has significantly better metabolic ability than any other organs. Similar views have also been mentioned in other studies^31^. We further analyzed the differential genes between the C1 and C3 subtypes and conducted GO functional enrichment analysis. Surprisingly, the results were mainly enriched in metabolism, confirming our previous view and reminding researchers that metabolic reprogramming by will be one of the promising directions for addressing liver cancer immunotherapy resistance.

We also developed a novel prognostic model and determined RiskScores through multivariate Cox regression analysis, comprising KPNA2, RRAGC, SPP1, FTCD, ADH4, and ANXA10. The transcriptional level of these key genes in tumor and normal cells is highly consistent with the risk coefficient trend. Interestingly, KPNA2 has been found to act as the upstream molecule of AKT signaling pathway to accelerate the HCC progression^32^. Guo et al. identified key pathways and genes associated with tumor recurrence in patients with HCC who had undergone liver transplantation, with a focus on the role of the immune system, including SPP1, one of the six promising genes mentioned above^33^. Moreover, the loss of FTCD was also reported to facilitate liver cancer occurrence through upregulating Peroxisome Proliferator Activated Receptor γ and Sterol Regulatory Element Binding Transcription Factor 2^34^. ANXA10 has also been revealed to be low expressed in liver cancer tissue, and its downregulation is related to the malignant phenotype of liver cells, vascular invasion, and HCC progression^35^.

Furthermore, we categorized patients with HCC into high-risk and low-risk groups based on their risk scores and examined their response to immunotherapy using TIDE software. Surprisingly, the low-risk group exhibited a lower TIDE score, indicating a higher likelihood of benefiting from immunotherapy. This underscores the efficiency of a prognostic model based on apoptotic gene sets in predicting immunotherapy benefits for patients with HCC. And then the effectiveness of our prognosis and immune response prediction model have been validated with good predictive ability through two immunotherapy datasets, GSE78220 and GSE135222.

Univariate and multivariate Cox regression analyses on RiskScore and clinical pathological data revealed RiskScore as the most significant prognostic factor, indicating its potential clinical utility. In contrast, some previously reported prognostic models for HCC exhibit instability, and certain clinical models are limited by overfitting.

## Conclusions

This study identified new HCC molecular subtypes based on apoptosis signaling pathway-related prognostic genes and established HCC prediction models using differentially expressed genes between these subtypes. The prognostic model has demonstrated effective and stable predictive performance for HCC outcomes and the ability to forecast immunotherapy benefits. Our risk score holds promising clinical application prospects, and further research on the model's constituent genes, including KPNA2, RRAGC, SPP1, FTCD, ADH4, and ANXA10, could shed light on the mechanism of immune escape, potentially improving the response rate of patients with HCC to immunotherapy.

## Methods

### Data collection

We acquired the most recent liver HCC sample data from The Cancer Genome Atlas (TCGA) database, which includes gene expression profiles from RNA-seq samples and information on single nucleotide variations (SNVs), which were analyzed using mutect2^36^. The latest HCCDB18 dataset was obtained from the HCCDB18 database. Clinical information was sourced from GSE14520 and GSE76427 datasets from the Gene Expression Omnibus. We sourced information about the Kyoto Encyclopedia of Genes and Genomes (KEGG) pathway “Apoptosis” and the corresponding genes from the Gene Set Enrichment Analysis (GSEA) website.

### Data processing

Processing TCGA-HHC RNA-seq data involved several key steps. Initially, we removed samples lacking follow-up information, followed by the elimination of samples without survival time or status data. We converted Ensemble IDs to Gene symbols and calculated the mean expression for multiple Gene Symbols. In the case of HCCDB18, an extra step was taken to exclude samples without expression profile data. Furthermore, when dealing with the datasets from GSE14520 and GSE76427, we adhered to a distinct set of steps. This includes the exclusion of samples lacking clinical follow-up, survival time, or status information, conversion of probes to gene symbols, removal of probes corresponding to multiple genes, and computation of the average expression for multiple gene symbols. These processes ensured that the data was properly prepared and standardized for subsequent analysis and interpretation.

### Single-sample GSEA (ssGSEA)

We used ssGSEA to evaluate the relative enrichment of the apoptosis signal pathway in each sample to assess its activation^37^. We ranked all genes in descending order of their expression levels and calculated the cumulative distribution function for genes with higher expression levels within the apoptosis-related gene set. Subsequently, we arranged all genes in the sample in the descending order of their expression levels and calculated the gene set enrichment score for each position. These scores were then averaged or weighted to derive the ssGSEA score for the sample, which provided an estimate of the relative abundance of the apoptosis signal pathway in both liver cancer and adjacent samples. Additionally, we utilized ssGSEA to analyze immune infiltration across different molecular isoforms.

### Cox regression analysis

Univariate Cox regression analysis assesses the impact of various factors on a variable, helping to determine whether a single factor significantly impacts the outcome variable. We used univariate Cox regression analyses on all 87 apoptosis-related genes, RiskScore, and clinical pathological features to identify significant prognostic factors in patients with HCC. Following multivariate Cox regression analysis, RiskScore and T stage emerged as the most significant prognostic factors. Moreover, we conducted a multi-factor COX regression analysis on the six genes comprising our prognostic model to determine the risk coefficient for each gene.

### SNV correlation analysis

In SNV analysis, we investigated mutations in both coding and non-coding regions. SNVs in the coding regions may affect protein sequence, while SNVs in the non-coding regions may influence gene expression and splicing. We examined the mutation frequencies of 13 genes with differential expression in cancer and adjacent tissues by analyzing four HCC samples sourced from the TCGA database.

### Consensus clustering and Kaplan–Meier (KM) survival analysis

We employed the ConsensusClusterPlus R package (v1.46.0) to cluster patient samples from the TCGA database in an unsupervised manner. Based on empirical cumulative distribution function plots, we determined that K = 3 represented the optimal cluster number. Additionally, we used the “survival” and “survminer” R packages to generate KM survival curves, allowing us to visualize survival data and outcomes within our study.

### Differential gene expression and gene ontology (GO) enrichment

We employed the limma package to identify significantly altered genes between C1 and C3 subtypes in both the TCGA and HCCDB18 datasets. Genes were filtered using a threshold of |log2FC|> 1 and a false discovery rate (FDR) < 0.05. The intersection of these two gene sets was determined utilizing a Venn plot. GO functional enrichment was performed on the 132 genes obtained through this process with WebGestaltR (V0.4.4).

### Least absolute shrinkage and selection operator (Lasso) regression analysis and development of the RiskScore model

Lasso regression analysis, a data mining technique, incorporates a penalty function into multivariate linear regression to optimize the model, mitigating issues like covariance and overfitting. When the coefficients reach zero, variable selection is achieved. We conducted Lasso regression utilizing the glmnet package. To develop our prognostic model, we performed tenfold cross-validation and calculated the confidence interval for each γ^16^. To compute the RiskScore for each patient, we used the following formula:

$$\mathrm{Riskscore }=\sum (\mathrm{\beta i}\times {\text{Expi}})$$

where Exp represents the expression of genes constituting the prognostic model, while β is the coefficient associated with each specific gene^38,39^.

### Human HCC tissue specimens

8 pairs of mankind HCC tissue and paired normal hepatic tissue were collected from the First Affiliated Hospital of Zhengzhou University, China. And the research protocols were accepted by the Ethics Committee of the First Affiliated Hospital of Zhengzhou University, China.

### Cell cultivation and real-time quantitative polymerase chain reaction (qPCR)

Both THLE2 human normal cell and Huh7 cells were obtained from the Henan Provincial Key Laboratory of Organ Transplantation (Zhengzhou, China) and cultivated in Dulbecco's Modified Eagle Medium with a high glucose concentration supplemented with antibiotics and fetal bovine serum under 5% CO_2_ and at 37 °C. Total RNA was extracted using reagents such as Trizol, chloroform, isopropanol, and ethanol. The extracted RNA was reverse transcribed into cDNA for qPCR analysis. The utilized primer sequences are listed in Table 1.

**Table 1:** The primers used in RT-qPCR.

Primer name	Primer sequence (5′–3′)
GAPDH-F	GGAGCGAGATCCCTCCAAAAT
GAPDH-R	GGCTGTTGTCATACTTCTCATGG
KPNA2-F	CTGCCCGTCTTCACAGATTCA
KPNA2-R	GCGGAGAAGTAGCATCATCAGG
RRAGC-F	TCGTTTCCAAAGGACTTCGGC
RRAGC-R	AAAAGAGGGTCTCGTTGGGTG
SPP1-F	GAAGTTTCGCAGACCTGACAT
SPP1-R	GTATGCACCATTCAACTCCTCG
FTCD-F	GGAATGCGTCCCCAACTTTTC
FTCD-R	TGTCGATAAGTCGGGAAGCTAC
ADH4-F	AGTTCGCATTCAGATCATTGCT
ADH4-R	CTGGCCCAATACTTTCCACAA
ANXA10-F	TTGTGGAGACTATGTGCAAGGA
ANXA10-R	GGTATGCCTCTGCAATCATCAT

### Tumor immune dysfunction and exclusion (TIDE)

The TIDE algorithm assesses how tumors hinder cytotoxic T lymphocyte function and infiltration, enabling immune escape and predicting the effectiveness of immune checkpoint inhibitors. We used the TIDE software to evaluate the efficacy of immunotherapy in high- and low-risk patient groups.

### Decision tree and decision curve analysis (DCA)

Decision tree analysis, a supervised machine learning classification method, can generate a decision tree model based on input data to predict or classify new data. In our study, decision tree analysis was carried out in R (version 4.0.2) to identify essential parameters for patient survival across distinct subtypes of HCC. The clinical value of a prognostic model is evaluated through statistical metrics like specificity, sensitivity, and the area under the Receiver Operating Characteristic (ROC) curve. However, these indicators do not consider the clinical utility of individual models. To address this, we used DCA, a tool commonly used for measuring clinical practicality, to estimate the reliability of our prognostic model.

### Supplementary Information

Supplementary Figure 1.

## Data Availability

Collections of data from this study are available in public databases. The newest expression and clinical follow-up data of HCC patients was obtained from TCGA database (https://www.cancer.gov/ccg/research/genome-sequencing/tcga). The latest HCCDB18 data set is obtained from HCCDB18 database (http://lifeome.net/database/hccdb/home.html). The GSE14520 and GSE76427 datasets were obtained from the gene expression omnibus (GEO) database (https://www.ncbi.nlm.nih.gov/geo/info/overview.html). The names of the repository/repositories are included in the article. Further inquiries can be directed to the corresponding author.
