# Supplementary material for: Novel molecular hepatocellular carcinoma subtypes and RiskScore utilizing apoptosis-related genes
Source: Sci Rep. 2024 Feb 16;14:3913. doi: 10.1038/s41598-024-54673-x (PMC10873508; doi:10.1038/s41598-024-54673-x)
Supplement: Supplementary file 1 — Supplementary Figure 1. [file 41598_2024_54673_MOESM1_ESM.docx]

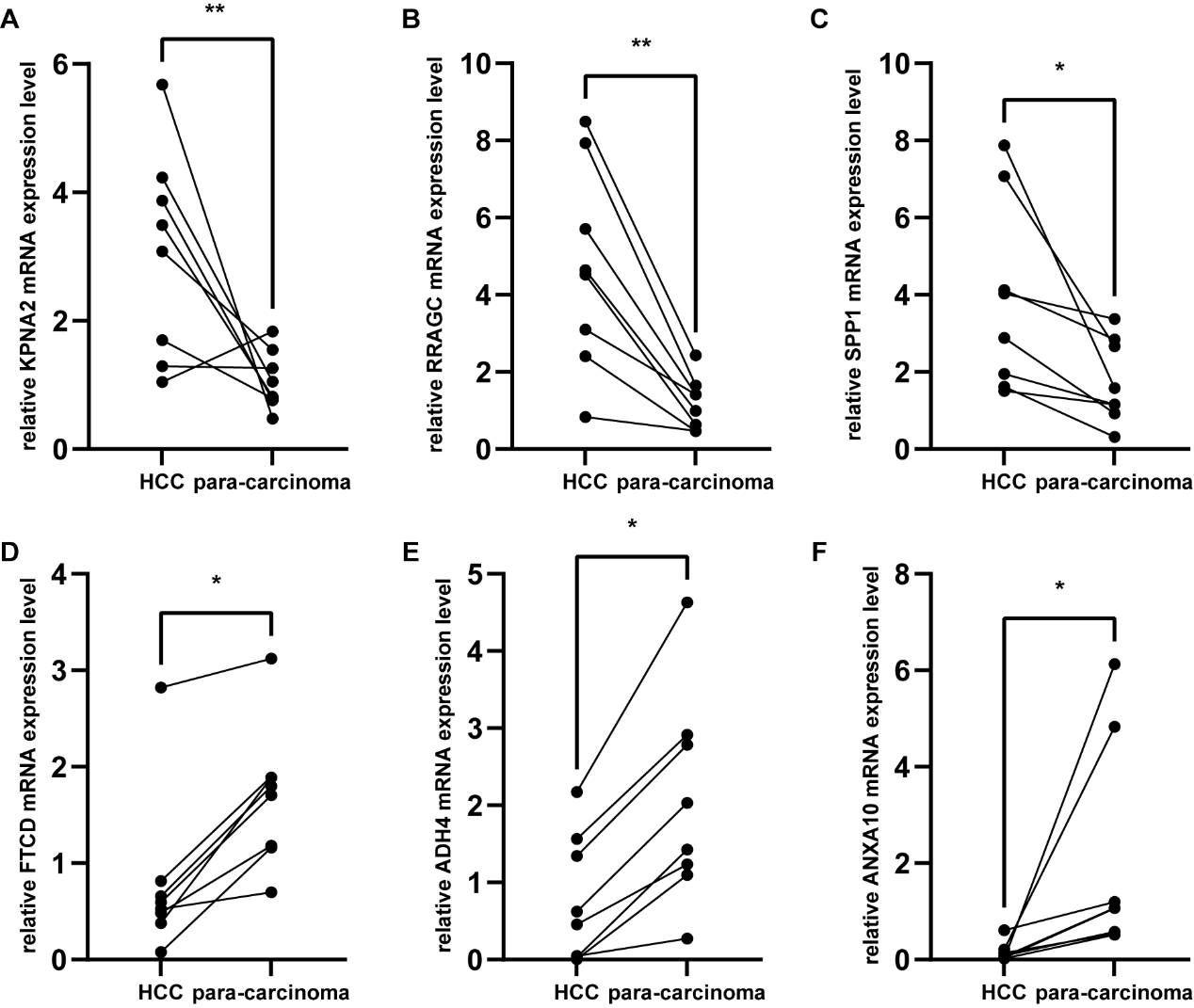


**Supplementary Figure 1.** (A-F) The differential expression of six genes (KPNA2, RRAGC, SPP1, FTCD, ADH4, and ANXA10) in HCC tissues and adjacent normal tissues.
